# Heat-Shock Mediated Overexpression of HNF1β Mutations Has Differential Effects on Gene Expression in the *Xenopus* Pronephric Kidney

**DOI:** 10.1371/journal.pone.0033522

**Published:** 2012-03-15

**Authors:** Kathrin Sauert, Stefan Kahnert, Magdalena Roose, Mazhar Gull, André W. Brändli, Gerhart U. Ryffel, Christoph Waldner

**Affiliations:** 1 Institut für Zellbiologie (Tumorforschung), Universitätsklinikum Essen, Essen, Germany; 2 Walter-Brendel-Zentrum für Experimentelle Medizin, Ludwig-Maximilians-Universität München, München, Germany; University of Colorado, Boulder, United States of America

## Abstract

The transcription factor HNF1B, encoded by the TCF2 gene, plays an important role in the organogenesis of vertebrates. In humans, heterozygous mutations of HNF1B are associated with several diseases, such as pancreatic β-cell dysfunction leading to maturity-onset diabetes of the young (MODY5), defective kidney development, disturbed liver function, pancreas atrophy, and malformations of the genital tract. The African claw frog *Xenopus laevis* is an excellent model to study the processes involved in embryogenesis and organogenesis, as it can be manipulated easily with a series of methods. In the present study, we overexpressed HNF1β mutants in the developing *Xenopus* embryo to assess their roles during organogenesis, particularly in the developing pronephric kidney. Towards this goal, we developed a heat-shock inducible binary Cre/loxP system with activator and effector strains. Heat-shock activation of the mutant HNF1B variants P328L329del and A263insGG resulted in malformations of various organs and the affected larvae developed large edemas. Defects in the pronephros were primarily confined to malformed proximal tubules. Furthermore, the expression of the proximal tubule marker genes tmem27 and slc3a1, both involved in amino acid transport, was affected. Both P328L329del and A263insGG downregulated expression of slc3a1. In addition, P328L329del reduced tmem27 expression while A263insGG overexpression decreased expression of the chloride channel clcnk and the transcription factor pax2. Overexpression of two mutant HNF1B derivatives resulted in distinct phenotypes reflected by either a reduction or an enlargement of pronephros size. The expression of selected pronephric marker genes was differentially affected upon overexpression of HNF1B mutations. Based on our findings, we postulate that HNF1B mutations influence gene regulation upon overexpression in specific and distinct manners. Furthermore, our study demonstrates that the newly established Cre/loxP system for *Xenopus* embryos is an attractive alternative to examine the gene regulatory potential of transcription factors in developing pronephric kidney as exemplified here for HNF1B.

## Introduction

The transcription factor HNF1B (vHNF1), encoded by the TCF2 gene, plays a crucial role in organogenesis of vertebrates. In humans, heterozygous mutations of HNF1B are associated with several diseases such as pancreatic β-cell dysfunction leading to maturity-onset diabetes of the young (MODY5), defective kidney development, disturbed liver function, pancreas atrophy, and malformations of the genital tract [1], [2]. Tissue-specific knock-outs in mice have verified these broad functions of hnf1b in organogenesis. The consequences of hnf1b deficiencies are manifested by polycystic kidney disease [3], pancreas agenesis with perturbed regional specification of the gut [4], defective insulin secretion [5], defective bile system morphogenesis with liver dysfunction [6], and altered liver bud formation in combination with abnormal gut regionalization [7]. The broad spectrum of hnf1b affected phenotypes is possibly also linked to its ability to interact with many other regulatory molecules [8]. Additionally, hnf1b is supposed to be a bookmarking factor necessary for the reopening of the chromatin of target genes after mitotic silencing [9]. Despite its many advantages for the analysis of hnf1b gene functions, the mouse model has also some limitations. Hnf1b-null mice die around embryonic day 7 due to a defect in visceral endoderm differentiation [10], [11] and a heterozygous mutation does not have any influence on development [11], [12]. Therefore, function of hnf1b was assessed by expressing Cre recombinase under the control of the kidney specific promoter Ksp-cadherin [3]. However, this promoter is under hnf1b control and thus deletes hnf1b only after an initial activation of hnfb1 [3]. Deletion of hnf1b prior to its activation has been achieved only most recently by tetraploid complementation of diploid knock-out embryos [13].

The African claw frog *Xenopus laevis* is an excellent model organism to analyze molecular processes involved in early embryogenesis and organogenesis [14]. The development of the embryos occurs outside of the mother and in the transparent larvae morphogenetic events can be monitored continuously. With regard to kidney development, *Xenopus* embryos and tadpoles are endowed with simple excretory organs, the pronephric kidneys. They are composed of four basic domains: proximal tubule, intermediate tubule, distal tubule and connecting tubule. These domains can be further subdivided into eight functionally distinct segments, which are surprisingly similar to those found in the murine kidney [15]. *Xenopus* embryos have therefore been employed to analyze the molecular properties of nine naturally occurring HNF1B mutations, which manifest as distinct renal diseases in humans [16], [17]. Injection of mRNA encoding distinct HNF1B mutants into *Xenopus* embryos broadly defined two distinct phenotype classes. The “enlargement class” represents HNF1B mutants that promote enlargement of the pronephric kidney upon overexpression. These mutants lack DNA binding capabilities and do not transactivate reporter gene expression in transfected cells. The A263insGG HNF1β mutant is the most potent one of this class. It carries a frameshift mutation leading to a truncated HNF1B protein lacking the transactivation domain and parts of the region involved in DNA binding [16], [18]. By contrast, overexpression of HNF1B mutants of the “reduction class” results in partial or complete agenesis of the pronephros. Typically, these mutants retain DNA binding capabilities. Most notably, overexpression of the P328L329del mutant in *Xenopus* embryos causes severe pronephric defects including agenesis [16]. The P328L329del mutant encodes a truncated HNF1B protein that retains both the dimerization and DNA binding domains and has an increased transactivation potential as revealed in cell culture experiments [16], [19]. In summary, the P328LP329del and A263insGG mutants interfere in distinct manners with pronephric kidney development in *Xenopus* embryos.

We have recently established a heat shock-inducible binary Cre/loxP system with activator and effector strains for the conditional overexpression of HNF1B mutants in the *Xenopus* embryos and tadpoles [20]. The conditional expression system allows activation of the Cre recombinase at any time during embryonic development. Furthermore, the system has the advantage that the Cre recombinase is only transcribed after the heat shock minimizing the potential adverse effects of constitutive Cre expression. Previously, we have demonstrated that heat-shock induced overexpression of the P328L329del mutant causes pronephros malformations and large edemas in *Xenopus* tadpoles [20]. We now define the developmental stages responsive to HNF1B mutant overexpression and describe the pronephric defects by using well-defined molecular markers that define distinct domains of the pronephric nephron [15], [21].

## Results

### A heat shock-inducible Cre/loxP system to conditionally overexpress HNF1B mutants

**Figure 1:**
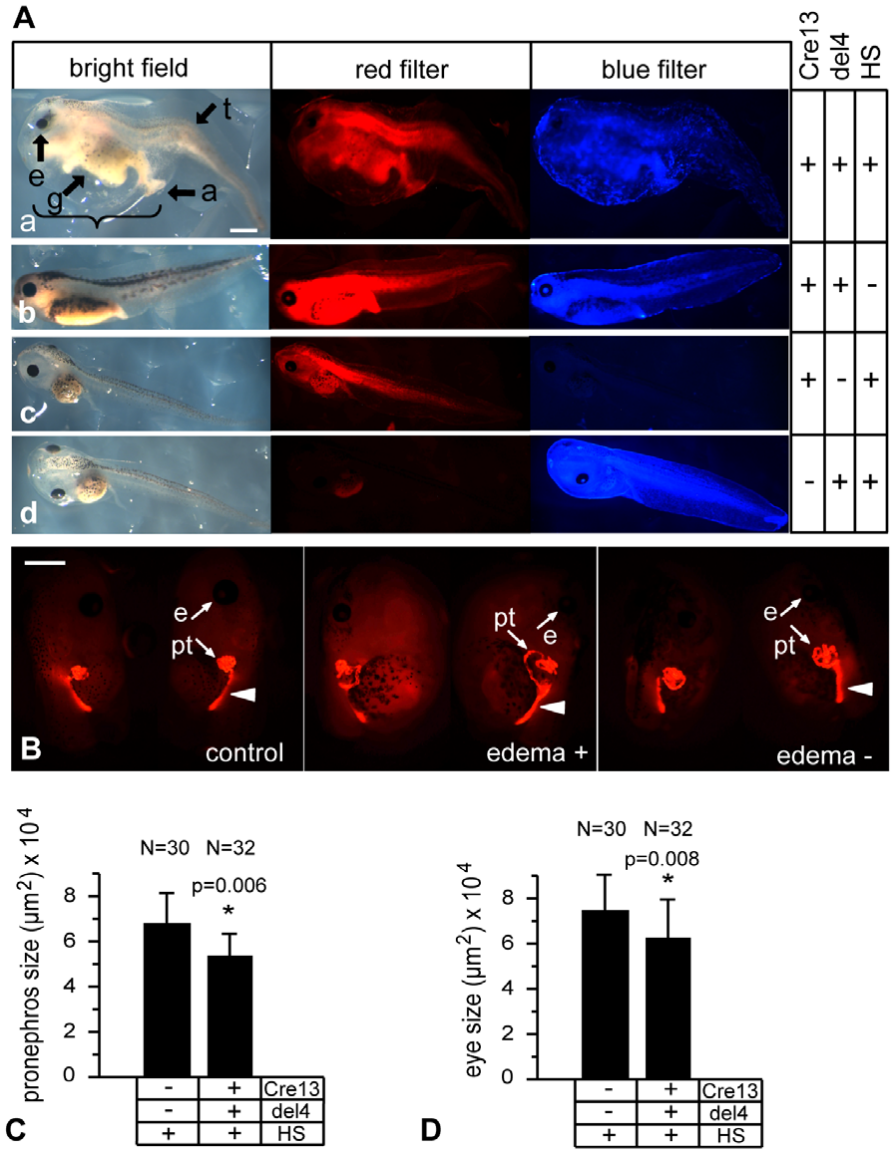
Effects of gastrula-stage overexpression of the P328L329del HNF1β mutation on larval development. Representative larvae of crossings between heterozygous HSPCre13 males and heterozygous 328del4 females. The F1 offspring were heat-shocked for two hours at 34°C at early gastrula (stage 11). Embryonic development was monitored until the larvae were free swimming at stage 41–46. A: (a) Representative deformed larva after heat-shock activated overexpression of the P328L329del mutation. (b): Double-transgenic control larva without heat-shock treatment (HS). (c) and (d): Control larvae lacking either the effector (c) or the activator (d) transgene. Black arrows indicate misformed structures and tissues. Abbreviations: g: abnormal gut, a: thickened anus, e: smaller eye, t: abnormal tail, bracket: edema. Red fluorescence: HSPCre13 positive animals. Blue fluorescence: 328del4 positive animals. B: Pronephros defects caused by overexpression of the P328L329del HNF1β mutation. Larvae were immunostained with a mixture of the 3G8 and 4A6 antibodies to visualize the entire pronephric kidney. For each larvae, both pronephric kidneys are shown. Mutant animals with or without edemas developed malformations of the pronephros and the eyes. Abbreviations: e, eye; pt, proximal tubules. White arrowheads indicate more distal parts of the pronephric kidney (pronephric duct). C and D: Quantitation of the average pronephric kidney (C) and eye sizes (D) in mutant and control animals. * = p<0.01 (Student's t-test). N refers to the number of single pronephri or eyes that were measured. Scale bar = 1 mm.

We have used a heat shock-inducible Cre/loxP system (Fig. S1) to conditionally overexpress the P328L329del and A263insGG HNF1B mutants in developing *Xenopus* embryos as described previously [20]. The Cre recombinase gene in the HSPCre13 strain is linked to the red fluorescent tdTomato marker gene permitting easy identification of Cre positive animals (Fig. 1A). We crossed heterozygous *Xenopus* HSPCre13 activator males with either heterozygous 328del4 or A263ins6 females to overexpress the P328L329del or A263insGG mutants, respectively, in their transgenic offspring (Fig. S1B, C).

### Effects of overexpressing the P328L329del HNF1B mutant in *Xenopus* embryos

F1 double transgenic offspring were heat-shocked for 2 hours at 34°C once they reached early gastrula (stage 11) and the embryonic development was monitored continuously until the larvae were free-swimming. 81% (N = 16) of the double transgenic animals overexpressing the P328L329del mutation (also referred to as “mutants”) had large edemas, smaller eyes, and massive malformations of the gut, stomach, and tail (Fig. 1Aa). These phenotypes became apparent starting from the late tailbud stages onwards. Double-transgenic animals, which had not been subjected to heat shock, developed normally with no apparent defects (Fig. 1 Ab). This was also true for heat-shocked animals lacking either the activator or effector transgenes (Fig. 1A c, d). The tight control of the HNF1B transgene in response to the heat-shock at this early developmental stage is shown in Fig. S2. The double transgenic larvae were immunostained to visualize the morphology of the pronephric kidneys (Fig. 1B). We observed malformations of the pronephric kidneys in mutant larvae compared to the control groups. The average size of the pronephric kidneys was reduced (Fig. 1C). Malformations were mainly associated with the proximal tubules, but some of the mutant larvae also displayed defects affecting more distal parts of the pronephric kidneys. Furthermore, the mutant larvae had smaller eyes (Fig. 1D). Taken together, overexpression of the P328L329del mutant in gastrula-stage *Xenopus* embryos has profound effects on pronephric kidney development.

### Defining the critical time window for pronephric kidney defects in *Xenopus* embryos overexpressing HNF1B mutants

**Figure 2:**
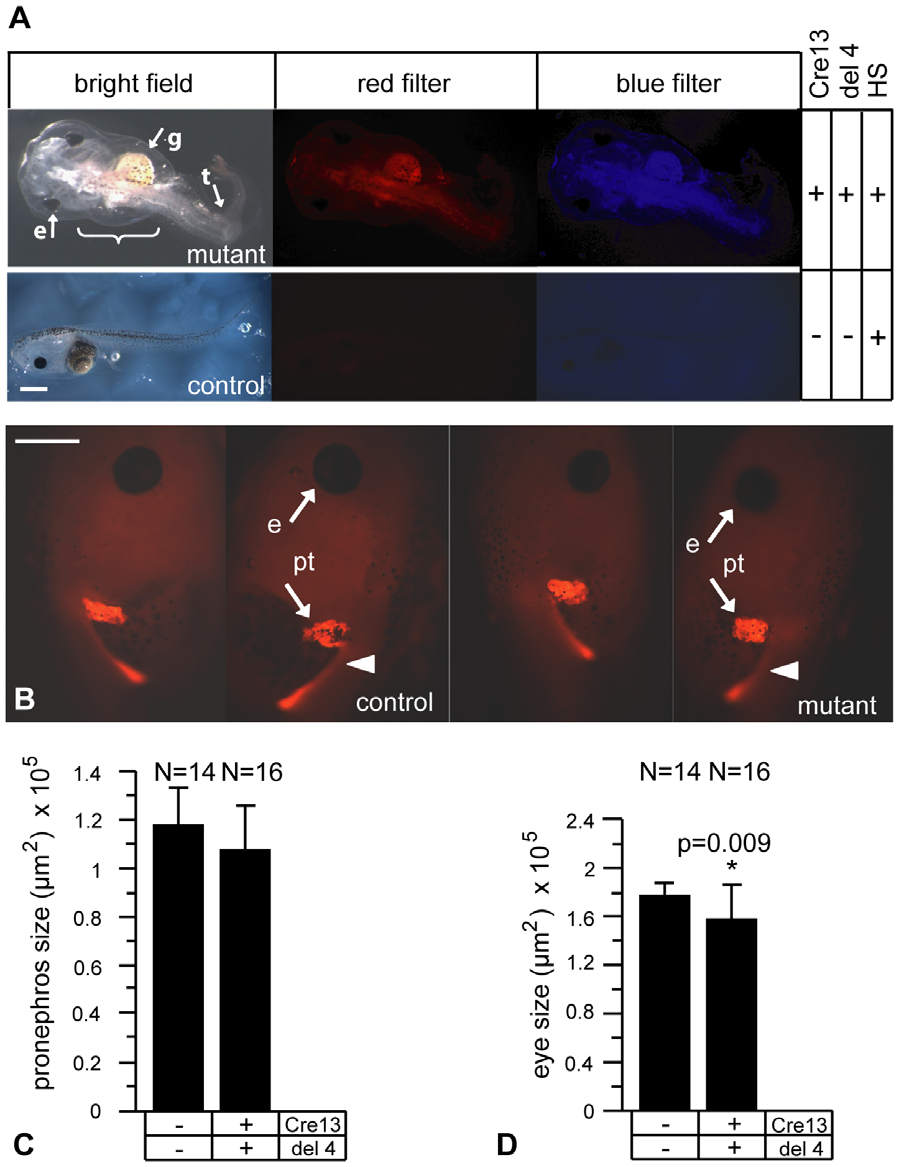
Effects of neurula-stage overexpression of P328L329del HNF1β mutation on larval development. Representative larvae of crossings between heterozygous HSPCre13 males and heterozygous 328del4 females. The F1 offspring were heat-shocked for two hours at 34°C at neurula (stage 19). A: Comparison of a larva with overexpressing the P328L329del HNF1β mutation with a non-transgenic control. Abbreviations: g, abnormal gut; e, reduced eye; t, abnormal tail; bracket: edema. Red fluorescence: HSPCre13 transgene. Blue fluorescence: 328del-4 transgene. HS: heat-shock treatment. The development of control larvae lacking either the effector or the activator transgene was not affected (data not shown). B: Analysis of the pronephric kidneys after overexpression of P328L329del HNF1β mutation at neurula stage. Mutant animals developed malformations of the eyes. Abbreviations: e, eye; pt, proximal tubules. White arrowheads indicate more distal parts of the pronephric kidney (pronephric duct). C and D: Quantitation of the average pronephric kidney (C) and eye sizes (D) in mutant and control animals. * = p<0.01 (Student's t-test). N refers to the number of single pronephri or eyes that were measured. Scale bar = 1 mm.

Next, we carried out heat-shock activation of the P328L329del mutant at neurula (st. 19) and tailbud (st. 25) stages in F1 double transgenics to determine the embryonic stages critical for the observed developmental phenotypes after HNF1B mutant overexpression. Heat-shock activation of the P328L329del mutant at neurula stage resulted in malformation of the intestine and tail in 80% (N = 10) of the mutant embryos (Fig. 2A). Edemas were only present in 30% of the mutant larvae. Interestingly, pronephros development appeared to be unaffected by overexpression of the P328L329del mutant (Fig. 2B, C). Similar to overexpression in gastrulae, the average eye size was significantly reduced when the overexpression was done in neurula stage embryos (Fig. 2D). Finally, when overexpression of the P328L329del mutant was initiated at the tailbud stage, no apparent developmental defects were observed (Fig. S3A–C). We conclude that pronephric kidney development is most impaired, if P328L329del overexpression is initiated early during gastrulation.

### Effects of HNF1B P328L329del overexpression on terminal differentiation of the pronephric kidney

**Figure 3:**
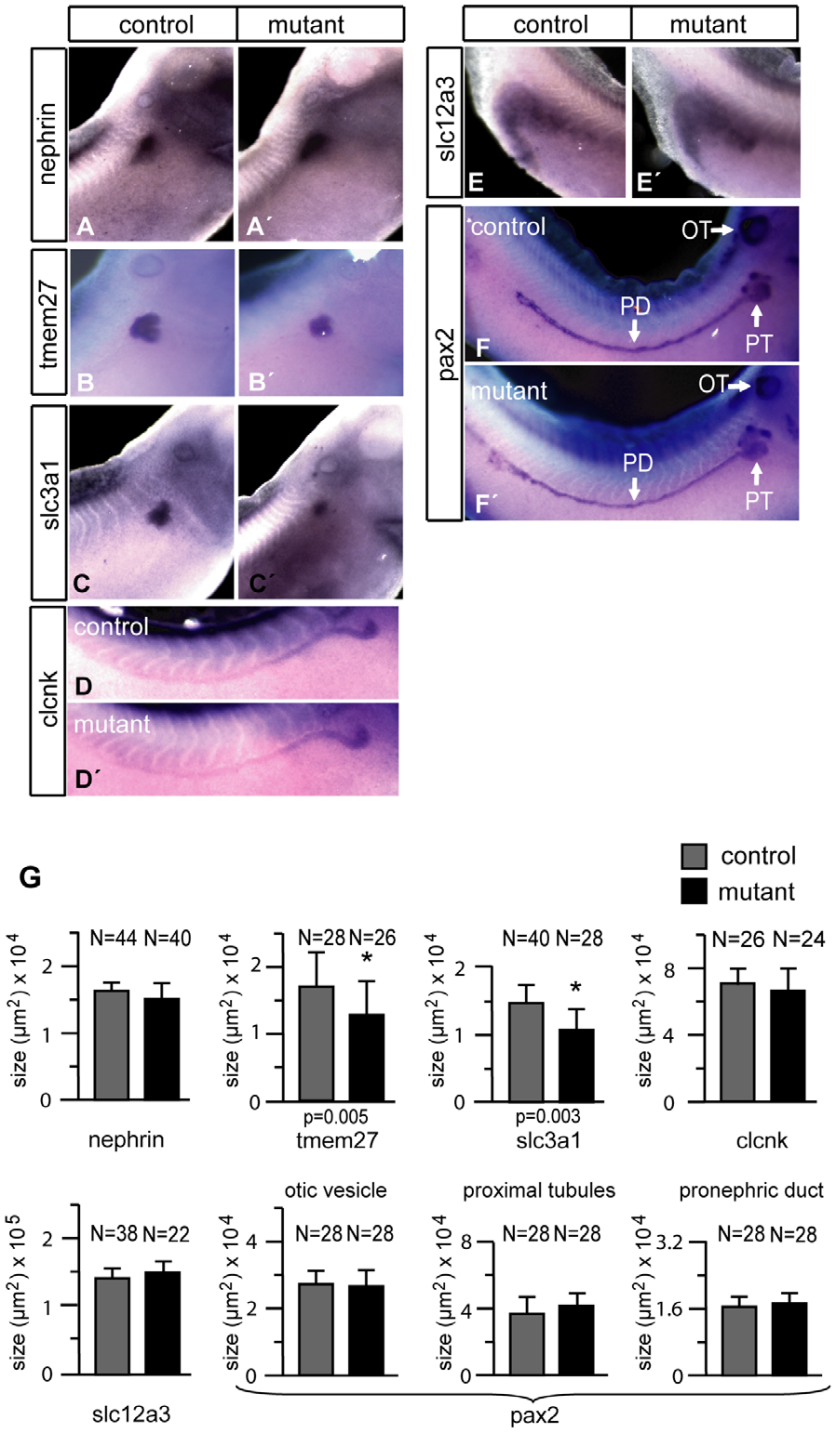
Analysis of pronephric marker gene expression in larvae overexpressing P328L329del HNF1β mutation. Whole mount *in situ* hybridization of non-transgenic controls (A–F) and mutant animals (A′–F′) heat-shocked at early gastrula (stage 11) were performed for the indicated markers. Abbreviations: OT: otic vesicle: PT, proximal tubules; PD: pronephric duct; G: Quantitation of the marker gene expression domains in mutant and control animals. * = p<0.01 (Student's t-test). N refers to the number of measurements performed.

**Figure 4:**
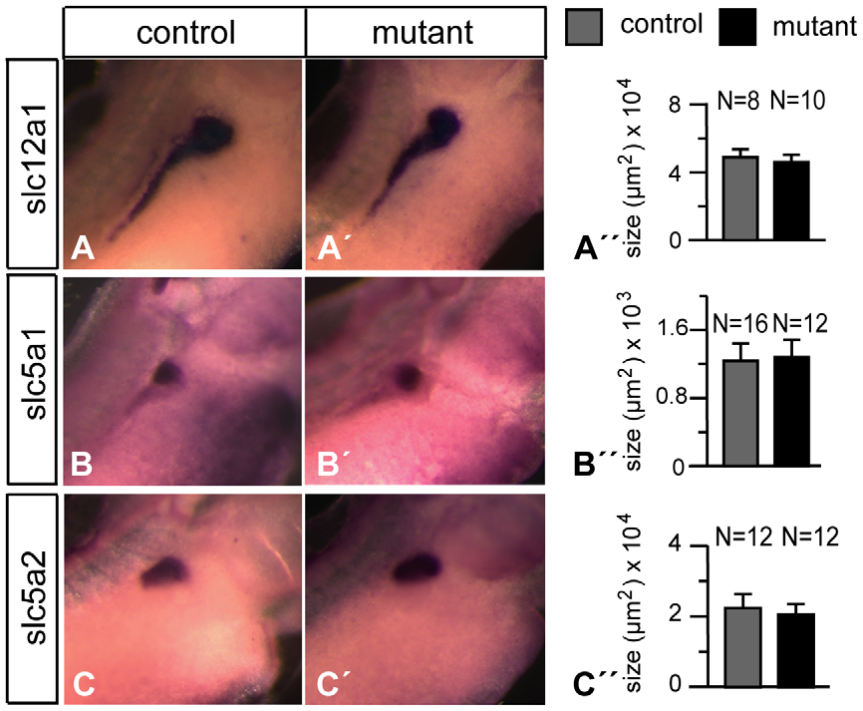
Analysis of nephron segment marker gene expression in larvae overexpressing P328L329del HNF1β mutation. Whole mount *in situ* hybridization of non-transgenic controls (A–C) and mutant animals (A′–C′) heat-shocked at early gastrula stage were performed for the indicated marker genes. A″–C″: Quantitation of the marker gene expression domains in mutant and control animals. * = p<0.01 (Student's t-test). N refers to the number of measurements performed.

We used a set of terminal differentiation markers to define more precisely the effects of P328L329del overexpression on the pronephric kidneys in mutant embryos. The marker genes were specific for different compartments of the pronephric kidney such as the podocytes, proximal tubules, intermediate tubules, distal tubules, and the cloaca. The analysis by whole mount in situ hybridization was conducted in stage 35/36 embryos, where the pronephric structures and nephron segments can be easily visualized without the need for sectioning of the stained embryos [15]. We observed identical hybridization signals with the podocyte marker nephrin in animals overexpressing the P328L329del mutant and non-transgenic controls (Fig. 3A′ and G). By contrast, the expression of the two proximal tubule markers tmem27 and slc3a1 (rBAT) were moderately reduced in mutant embryos (Fig. 3B′, C′ and G). Next, we used the chloride channel clcnk to probe the intermediate, distal and connecting tubule [15], but failed to see diffences between P328L329del mutants and controls (Fig. 3D′ and G). Similarly, expression of the Na-Cl transporter slc12a3, a marker of the distal tuble segment 2 (DT2), connecting tubule, and the cloaca [15], and the transcription factor pax2, present in all epithelia of the pronephros [22], were unaffected (Fig. 3E′ and G, F′ and G). Finally, we analyzed the pronephric kidneys of mutant larvae for expression of three additional marker genes (slc12a1, slc5a2 and slc5a1) to investigate subtle effects of P328L329del overexpression on patterning and/or terminal differentiation of the pronephric kidney. The Na-K-Cl transporter slc12a1 is expressed in the intermediate tubule segments IT1 and IT2 as well as in the distal tubule segment DT1 [15]. Consistent with the results for clcnk, expression of slc12a1 was unaltered in mutant larvae (Fig. 4A). Given the altered morphology of proximal tubules (Fig. 1B) and the downregulation of tmem27 and slc3a1 (Fig. 3B′, C′), we asked whether expression of other marker genes of the proximal tubule would be altered similarly. Expression of the Na-glucose cotransporter slc5a2 is confined to proximal tubule segments PT1 and PT2, whereas slc5a1 marks PT2 and PT3 [15]. *In situ* hybridization analysis failed however to reveal significant differences between animals overexpressing the P328L329del mutant and non-transgenic controls (Fig. 4B, C). We conclude that overexpression of the “reduction class” HNF1B mutation P328L329del does not interfere with patterning and terminal differentiation of the pronephric kidney, but reduces the expression of the proximal tubule marker genes tmem27 and slc3a1 indicating a possible effect on amino acid uptake.

### Distinct effects of HNF1B A263insGG overexpression on *Xenopus* pronephros differentation

**Figure 5:**
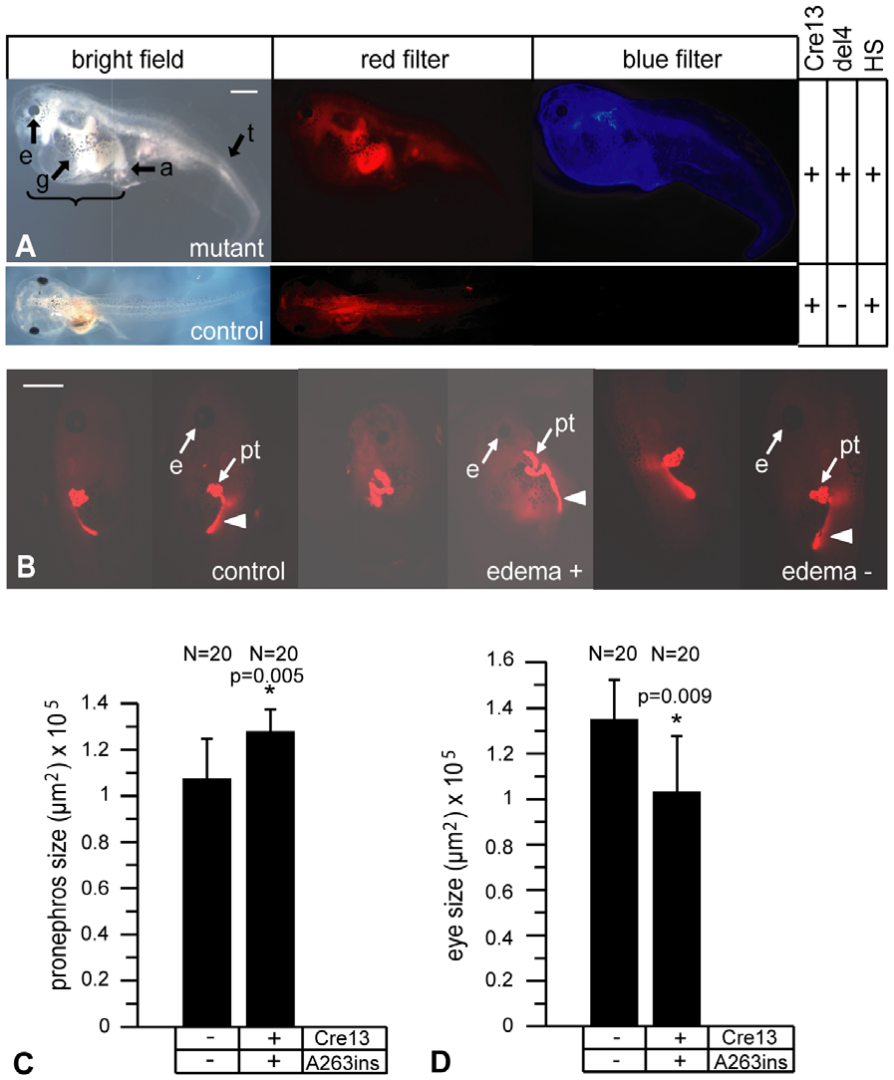
Effects of overexpressing the A263insGG HNF1β on larval development. Representative larvae of a crossing between heterozygous HSPCre13 males and heterozygous A263ins6 females. The F1 offspring were heat-shocked for two hours at 34°C at early gastrula (stage 11). A: Comparison of a larva overexpressing the A263insGG HNF1β mutation with a non-transgenic control. Abbreviations: a: thickened anus; g: abnormal gut; e: reduced eye; t: abnormal tail; bracket: edema. Red fluorescence: HSPCre13 transgene. Blue fluorescence: 328del4 transgene. HS: heat-shock treatment. B: Analysis of the pronephric kidneys overexpressing the A263insGG HNF1β mutation Mutant animals with or without edemas developed malformations of the pronephric kidneys and the eyes. Abbreviations: e: eye; pt: proximal tubules. White arrowheads indicate more distal parts of the pronephric kidney (pronephric duct). C and D: Quantitation of the average pronephric kidney (C) and eye sizes (D) in mutant and control animals. * = p<0.01 (Student's t-test). N refers to the number of single pronephri or eyes that were measured. Scale bar = 1 mm.

The HNF1B mutant A263insGG encodes a truncated HNF1B protein lacking the transactivation domain and parts of the region involved in DNA binding [18] and belongs to the “enlargement class” of HNF1B mutants. We crossed heterozygous HSPCre13 males with heterozygous females of the A263ins-6 effector strain to explore the effects of A263insGG overexpression in *Xenopus* embryos. Heat-shock activation to induce overexpression of the A263insGG mutant was done at early gastrula (stage 11) as the analysis of P328L329del revealed the most prominent effect of HNF1B on pronephros development at this stage. Using this protocol we observed that 50% (N = 10) of the double transgenic F1 offspring developed large edemas and malformations of the gut, stomach, and tail were evident (Fig. 5A). The mutant animals had also significantly smaller eyes and the pronephric kidneys were malformed (Fig. 5B, D). The pronephric defects were characterized by dilated proximal tubules and, in some cases, more distal parts of the pronephric kidney were also affected (Fig. 5B). Interestingly and in contrast to animals overexpressing the P328L329del mutant, the average pronephros size was significantly enlarged in animals overexpressing the A263insGG mutant (Fig. 5C).

**Figure 6:**
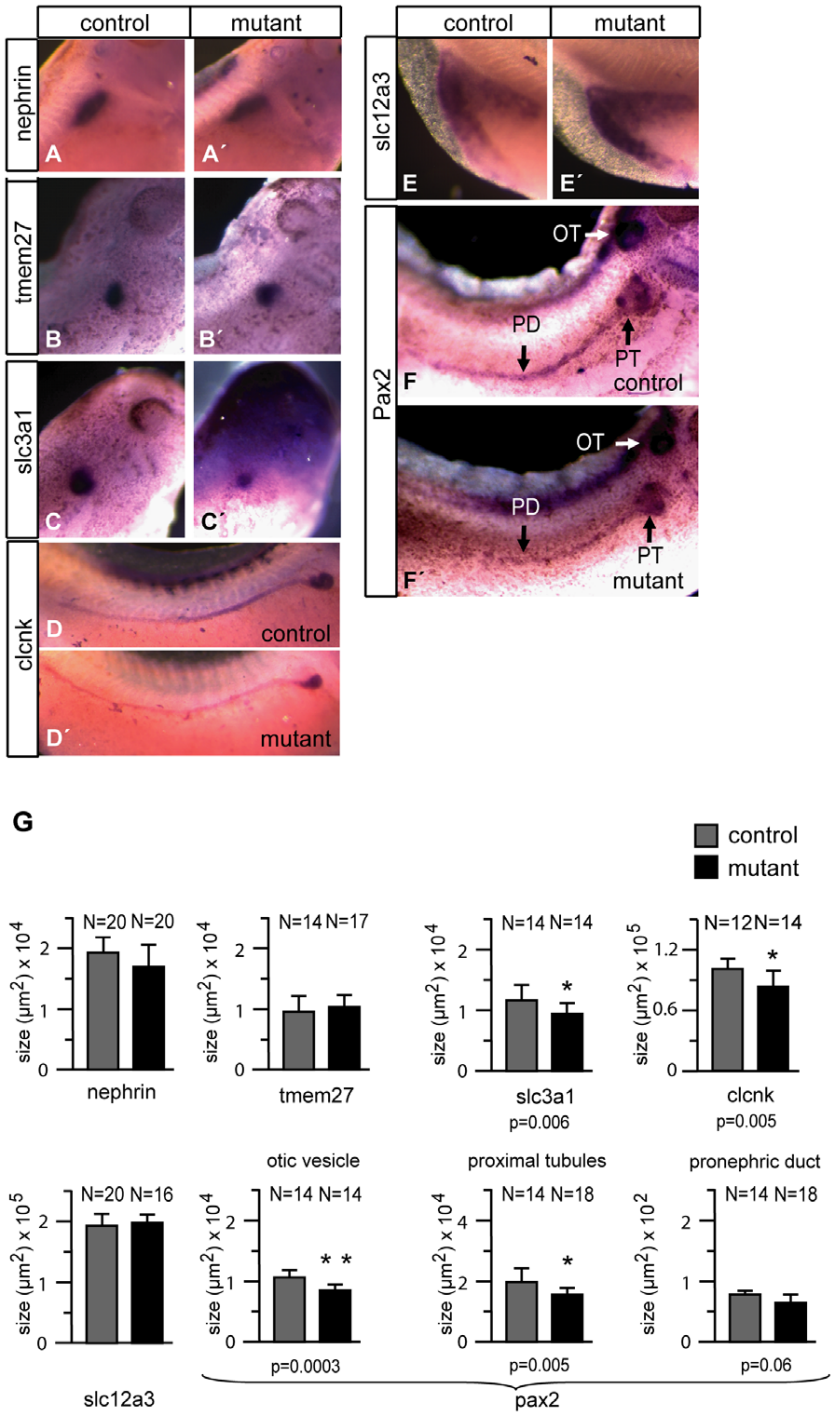
Analysis of pronephric marker gene expression in larvae overexpressing A263insGG HNF1β mutation. Whole mount *in situ* hybridization of non-transgenic controls (A–F) and mutant animals (A′–F′) were performed for the indicated markers. G: Quantitation of the marker gene expression domains in control and mutant animals. * = p<0.01 ** = p<0.001 (Student's t-test). N refers to the number of measurements performed.

We investigated next the expression of various pronephric marker genes by *in situ* hybridization. Expression of the podocyte marker nephrin was comparable in mutant and non-transgenic control animals (Fig. 6A, A′, G). Similarly, expression of tmem27 (Fig. 6B, B′, G) and slc12a3 (Fig. 6E, E′, G) was unaffected. By contrast, we noted moderate downregulation of slc3a1 (Fig. 6C, C′, G) and clcnk (Fig. 6D, D′, G) expression in the mutants. Furthermore, we noted that the expression of pax2 was also affected in mutant larvae with activated A263insGG (Fig. 6F, F′, G). We compared the expression levels of pax2 in greater detail by quantifying the gene expression in the proximal tubule, the pronephric duct (comprising the intermediate, distal and connecting tubules [15]), and the otic vesicle. While pax2 expression in the mutant larvae was not affected in the pronephric duct, it was significantly decreased in both the proximal tubule and the otic vesicle (Fig. 6G). In summary, overexpression of the A263insGG HNF1B mutant does not impair pronephric kidney morphogenesis, but has subtle effects on the expression of selected pronephric marker genes, which are distinct from those observed in animals overexpressing the P328L329del HNF1B mutant.

## Discussion

In this study we have applied the binary Cre/loxP system [20] to conditionally overexpress the mutated HNF1B derivatives P328L329del and A263insGG in the developing *Xenopus* embryo. Using this approach we confirmed our previous results based on RNA injection experiments [17] that these mutants interfere with kidney development by either enhancing or reducing the size of the pronephros. In contrast to the RNA experiments the effects observed now were much more homogenous, possibly reflecting the fact that in RNA injections the amount of overexpressed HNF1B mutants differs from one animal to the next quite extensively. The homogenous response using the Cre/loxP system has allowed us now to analyze in detail the effects of the mutant HNF1B on pronephros differentiation. In our transgenic Cre/loxP technique we observed in general a much broader effect. This is partly due to the overexpression in the entire animals, whereas RNA injections were restricted to half of the injected embryo. Therefore, in the Cre/loxP system the HNF1B mutants displayed large edemas, most likely because the pronephros is not developed properly on both sides, as seen in particular by the malformed proximal tubules (Fig. 1 and 5). We assume that this organ cannot adopt its excretory function. This would be similar to the kidney specific hnf1b manipulation in mice that leads to polycystic kidneys [3], [23] and to mutation of the hnf1b gene in zebrafish that is reflected in kidney defects, especially the formation of cysts [24]. The observed kidney defects are consistent with the fact that hnf1b is expressed in the Xenopus pronephros anlage [8], [25] arguing for a possible interaction of the mutant with the endogenous hnf1b.

We assume that the extensive edema formation reflects malfunction of the excretory system, but the consequence of impaired cardiovascular and lymphatic functions cannot be excluded [26], as the heat-shock treatment leads to a global overexpression of mutated HNF1B that may affect morphogenesis of lymph and/or blood vessels [26]. Beside the kidney defects we observed many other malformations. Some of them reflect organs expressing hnf1b. This is true for the gastrointestinal tract known to express hnf1b [27]. In these organs overexpression of the mutants leads to thickening of the anus and lack of coiling of the gut. Moreover, in *Xenopus* embryonic development HNF1β is expressed in the tailbud [8] and this finding possibly explains the observed defects in tail formation upon heat-shock activation of the mutant. In contrast, the reduced eye size observed in the mutants (Fig. 1 and 5) is unexpected, as hnf1b is not expressed in the eye [8]. However, it might be possible that it is due to an interaction with DCoH, a cofactor of HNF1B [28], that is expressed in the eye in the absence of HNF1B and interacts with the dimerization domain of HNF1B (Fig. S1B). Thus, we postulate that ectopic expression of HNF1B mutants inhibits DCoH function in the eye [29]. Alternatively, the eye phenotype might also be of secondary origin, as at least in zebrafish [24] and mice [10] hnf1b participates in patterning of the hindbrain that may influence eye formation. A secondary effect might also be due in the otic vesicle that lacks hnf1b expression, since the hindbrain is involved in the induction of the otic vesicles [30]. Accordingly, a homozygous mutation of hnf1b affects the patterning of the otic vesicle in the zebrafish [31]. Therefore, we assume that the smaller otic vesicle upon A263insGG overexpression in *Xenopus* represents such an impaired patterning event.

Performing heat-shock activation of P328L329del at various stages we observed that the early gastrula stage is most sensitive, whereas less dramatic effects were seen in the neurula and upon heat-shock activation at tailbud stage the larvae appeared normal. This high sensitivity in early development might be expected for a factor involved in organogenesis and agrees with the mouse model where inactivation of hnf1b from postnatal day 10 onward failed to elicit cystic dilatations in the proximal tubules [9].

Analyzing a whole set of markers specific for nephron segments by in situ hybridization we did not observe any effect on the patterning and terminal differentiation of the pronephric kidney, but some moderate effect on defined markers. Most notably, upon P328L329del overexpression the analysis of selected pronephric terminal differentiation markers revealed that exclusively the proximal tubule markers slc3a1 and tmem27 were affected. Our data in *Xenopus* imply that these two proteins are HNF1β target genes, a finding that might be also relevant for the human system. slc3a1, also known as rBAT, is a member of the solute carrier gene family of transporters. These proteins are essential for the transport of organic molecules, especially amino acids. Together with slc7a9, slc3a1 represents the rBAT/b^0,+^AT transporter that is primarily localized in the epithelial cells of the proximal tubules [32]. It is essential for the renal reabsorption of the amino acid cystin [33] and mutations in slc3a1 or slc7a9 interfere with the function of the rBAT/b^0,+^AT transporter. Thus, the reabsorption function of the kidney for amino acids, most notably cystin, is impaired leading to cystinuria. In human patients cystinuria is attended by mutations in SLC3A1 and SLC7A9 [34], but it is not known whether these defects are present in carriers of HNF1β mutation. Further support that slc3a1 is an HNF1B target gene is given by potential binding sites for HNF1 in the SLC3A1 promoter [35]. The second gene we propose to be regulated by HNF1B, tmem27, has in fact recently been identified as a target gene of the HNF1 complex [36] and is indispensable for the renal reabsorption of amino acids [37]. Interestingly, tmem27 colocalizes with slc3a1 in the epithelial cells of the proximal tubules and regulates amino acid transport by influencing transporters such as rBAT/b^0,+^AT [38], [39]. This implies HNF1B regulation of several components of a specific transporter complex. We speculate that the impaired function of this complex ultimately results in dilated proximal tubules and large edemas in Xenopus. A third potential HNF1B target gene is clcnk whose expression was decreased upon A263insGG overexpression. Its human orthologues, CLCKA and B, are specifically expressed in the kidney and have crucial roles in the reabsorption of Na+ and Cl− in the distal tubules and in urine concentration [40], [41]. Both genes are involved in the pathophysiology of Bartters syndrome types III and IV [42] and renal diabetes insipidus [43], but both these diseases are not known to be included in patients with HNF1B deficiency.

The developmental defects of the gastrointestinal tract, tail and eye observed upon heat-shock activation of A263insGG were very similar to the ones observed for P328L329del. But in contrast to the significant reduction of pronephros size upon P328L329del overexpression, heat-shock activation of A263insGG resulted in a significantly enlarged pronephros. This distinct phenotype of the two mutants made previously by RNA injections [17] we could now extend to the molecular level. We show that both mutants interfere with slc3a1 expression, whereas P328L329del exclusively impairs tmem27 function and A263insGG specifically downregulates clcnk as well as pax2. This clear difference between the mutants A263insGG and P328L329del may not be too surprising, since these deletion mutants retain different protein domains (Fig. S1B) and have distinct molecular properties [17]. Although in humans the A263insGG [18] and P329L329del [44] mutant carriers have been described with slightly different phenotype, these potential differences cannot be validated, as each mutant has been identified in only one family. Taken together our findings reveal that the Cre/loxP system we have developed in *Xenopus* is an attractive novel alternative to dissect the potential of regulatory factors as exemplified here for HNF1B in kidney physiology.

## Materials and Methods

### Animals

Husbandry, breeding and hormone injections of *Xenopus* were approved by the local veterinary authorities (Landesamt für Natur-, Umwelt-, und Verbraucherschutz, Nordrhein- Westfalen, Permit No. B 952/07). The transgenic strain A263ins6 was established as described for 328del4 [20].

### Plasmids

Plasmid pLCMV:ECFP(loxP)(FRT)HNF1βP328L329del or –A263insGG (Addgene plasmid 31440) is composed of the CMV-driven ECFP gene flanked by loxP and FRT sites followed by the open reading frame of the human HNF1β sequence containing the mutant P328L329del or A263insGG [16], [17].

### Heat-shock treatment

Embryos were obtained from *in vitro* fertilizations. They were kept at 16°C prior to heat-shock treatment to prevent premature activation of the HSP70 promoter. Heat-shock treatments were performed at 34°C for 2 hrs by transferring the embryos with a sieve into a water bath. In comparison to the original protocol [20], the heath-shock treatment was extended from one to two hours as this resulted in more robust and homogenous activation of transgene expression (unpublished data). After heat-shock treatment, the embryos were kept at 22°C. Developmental stages were determined according to Nieuwkoop and Faber [45].

### Immunohistochemistry and quantitation

Once developmental stage 46 was reached, the tadpoles were fixed and stained with the monoclonal antibodies 3G8 or 4A6 as described [46]. Differences in marker gene expression were quantified by measuring the size of the expression domains in the lateral views of mutant and control animals. Using AxioVision 4.6 software (Carl Zeiss Imaging Solutions) the outlines of the expression domains were marked and hereby the size of the area enclosed was measured automatically. The expression domain was defined by the widest diameter between the dorsal to the ventral borders of the immunostained pronephros, which included the pronephric tubules and anterior part of the pronephric duct. The measurements were performed using AxioVision 4.6 software (Carl Zeiss Imaging Solutions). Significant differences were scored using the Student's t-test to calculate p-values.

### Quantitative RT-PCR

RNA from tail tips of single larvae was isolated with the peqGOLD Total RNA Kit (PeqLab). For cDNA synthesis the High Capacity cDNA Reverse Transcription Kit (Applied Biosystems) was used. SYBR-green real-time PCR was performed on a 7900HT Sequence Detection System (Applied Biosystems) using Power-SYBRGreen-Mix (Applied Biosystems). Templates were determined in duplicate. Results are normalized to ornithin decarboxylase (odc) expression levels. In all cases water only and reverse transcriptase negative controls failed to produce specific products. Primers used were HNF1B_RNA_FOR: 5′- GGGACGTCGGACGAAGCTT-3′ and HNF1B_RNA_REV: 5′- GCTTTTCGTCCATTAGCTTT-3′ for the exogenous P328L329del transcripts; ODC5: 5′-TGGGCTGGAATCGTATCGT-3′ and ODC6: 5′- CATTGAATGTCGAGGCTGCA-3′ to detect ornithin decarboxylase (odc) as a reference.

### *In situ* hybridizations

Whole mount *in situ* hybridization was performed using the standard technique with BM Purple staining [14]. The following plasmids were linearized and transcribed to generate antisense RNA probes: pCMV-Sport6-*collectrin*-SmaI/T7 [39], pBluescriptIISK+ - Pax2-EcoRI/T3 [22], pCMV-Sport6-Nephrin (GeneBank accession number: BI477611) -EcoRI/T7,pCMV-Sport6-*clcnk* (GeneBank Accession Number: NM_001085839) – SmaI/T7, pCMV-Sport6-*slc12a3* (GeneBank accession number: CA790325) – SmaI/T7, pCMV-Sport6-*slc3a1* (GeneBank accession number: BU903456) – EcoRI/T7. Plasmids and *in situ* hybridizations for slc5a1, slc5a2, and slc12a1 were performed as described previously [15]. Quantitation of the in situ hybridizations results were performed as described for *Immunohistochemistry*.

### Fluorescence microscopy

Fluorescence microscopy was done with a Leica MZ/FLIII stereomicroscope equipped with the appropriate filters as described previously [47].

## Supporting Information

Figure S1 **Cre activator and effector strains for the overexpression of HNF1β mutants.** A: Scheme of the construct present in the HSPCre13 activator strain. Located on the same plasmid, Cre recombinase is under the control of the HSP70 promotor, whereas expression of the tdTomato reporter gene is driven by the CMV promotor. B: Domain structure of HNF1β and its mutant derivatives P328L329del and A263insGG. POU_S_ and POU_H_ refer to the POU specific domain and POU homeodomain, respectively. C: Scheme of the transgenic effector constructs used in the present study. The CMV-driven blue fluorescent protein ECFP is used as a reporter. It is flanked by loxP sites (black triangles) followed by the open reading frame of HNF1β harboring either the P328L329del mutations (328del4 strain) or the A263insGG mutation (A263ins6 strain). Successful recombination of the effector construct upon HSPCre13 activation leads to the expression of either P328L329del or A263insGG. The effector strains contain in addition a FRT site, which is not shown here [20].
(TIF)

Figure S2 **Expression of P328L329del transcripts upon crossing the HSPCre13 with the 328del4 strain.** Quantitative RT-PCR analysis of a crossing between a heterozygous HSPCre13 male and a heterozygous P328L329 female. The F1 offspring were heat-shocked for two hours at 34°C at early gastrula (stage 11). Double transgenic larvae were identified by the presence of red and blue fluorescence at stage 40 (see Fig. S1). mRNA levels of P328L329del were analyzed by quantitative RT-PCR using specific primers (see Material and Methods) and the results were normalized to odc expression levels. The single bars represent the mean of N larvae +/− standard deviation. n.d. = no detection of P328L329del transcripts. E = edema M = malformations.
(TIF)

Figure S3 **Effects of tailbud-stage overexpression of P328L329del HNF1β mutation on larval development.** Representative larvae of crossings between heterozygous HSPCre13 males and heterozygous 328del4 females. The F1 offspring were heat-shocked for two hours at 34°C at tailbud stage (st. 25). A: Comparison of a larva with overexpressing the P328L329del HNF1β mutation with a non-transgenic control. Red fluorescence: HSPCre13 transgene. Blue fluorescence: 328del4 transgene. C and D: Quantitation of the average pronephric kidney (C) and eye sizes (D) in mutant and control animals. * = p<0.01 (Student's t-test). N refers to the number of single pronephri or eyes that were measured. Scale bar = 1 mm.
(TIF)
