# Supplementary figures and images for: Heat-Shock Mediated Overexpression of HNF1β Mutations Has Differential Effects on Gene Expression in the Xenopus Pronephric Kidney
Source: PLoS One. 2012 Mar 15;7(3):e33522. doi: 10.1371/journal.pone.0033522 (PMC3305329; doi:10.1371/journal.pone.0033522)

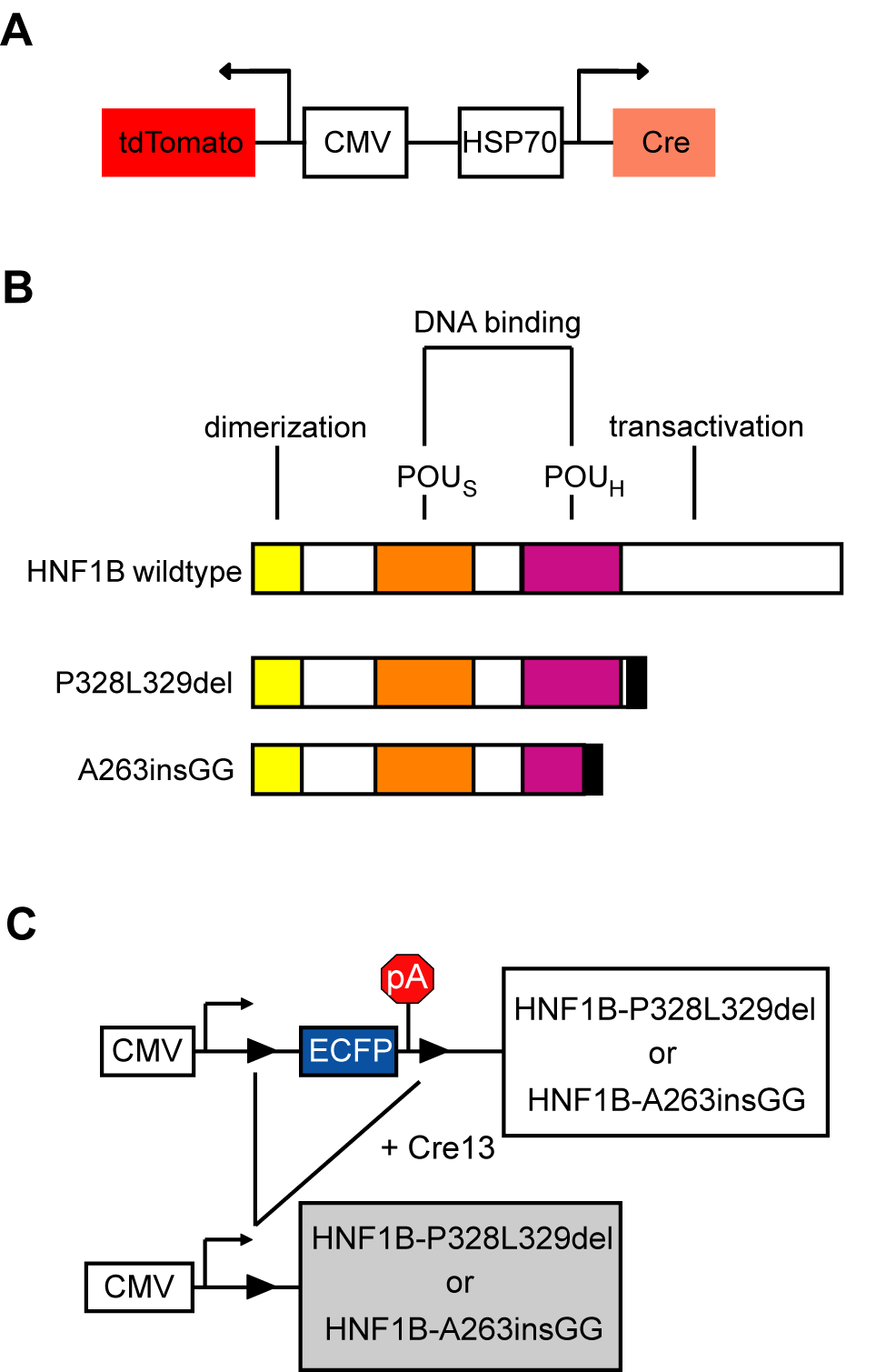

Supplement: Figure S1 — Cre activator and effector strains for the overexpression of HNF1β mutants. A: Scheme of the construct present in the HSPCre13 activator strain. Located on the same plasmid, Cre recombinase is under the control of the HSP70 promotor, whereas expression of the tdTomato reporter gene is driven by the CMV promotor. B: Domain structure of HNF1β and its mutant derivatives P328L329del and A263insGG. POUS and POUH refer to the POU specific domain and POU homeodomain, respectively. C: Scheme of the transgenic effector constructs used in the present study. The CMV-driven blue fluorescent protein ECFP is used as a reporter. It is flanked by loxP sites (black triangles) followed by the open reading frame of HNF1β harboring either the P328L329del mutations (328del4 strain) or the A263insGG mutation (A263ins6 strain). Successful recombination of the effector construct upon HSPCre13 activation leads to the expression of either P328L329del or A263insGG. The effector strains contain in addition a FRT site, which is not shown here [20]. (TIF) [file pone.0033522.s001.tif]

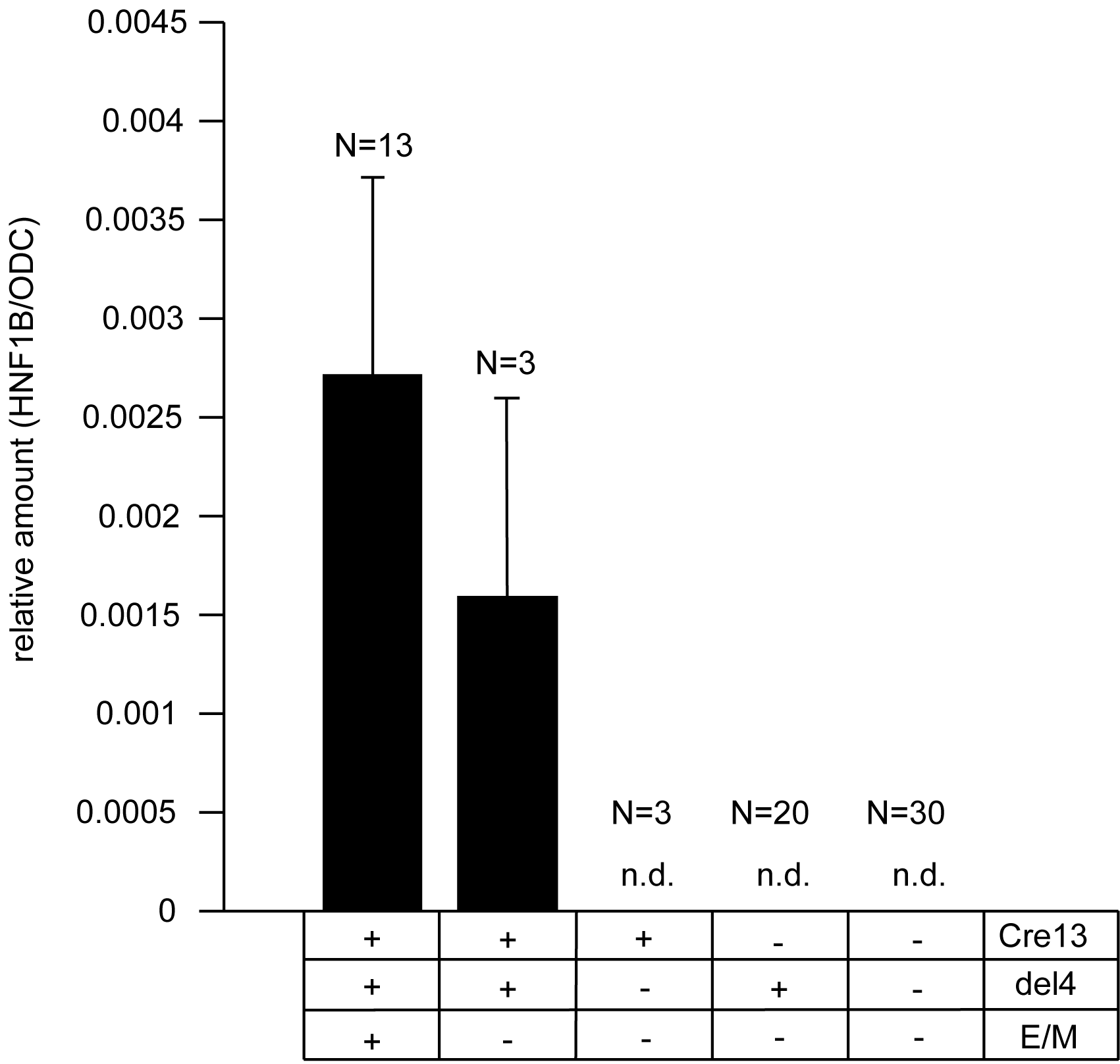

Supplement: Figure S2 — Expression of P328L329del transcripts upon crossing the HSPCre13 with the 328del4 strain. Quantitative RT-PCR analysis of a crossing between a heterozygous HSPCre13 male and a heterozygous P328L329 female. The F1 offspring were heat-shocked for two hours at 34°C at early gastrula (stage 11). Double transgenic larvae were identified by the presence of red and blue fluorescence at stage 40 (see Fig. S1). mRNA levels of P328L329del were analyzed by quantitative RT-PCR using specific primers (see Material and Methods) and the results were normalized to odc expression levels. The single bars represent the mean of N larvae +/− standard deviation. n.d. = no detection of P328L329del transcripts. E = edema M = malformations. (TIF) [file pone.0033522.s002.tif]

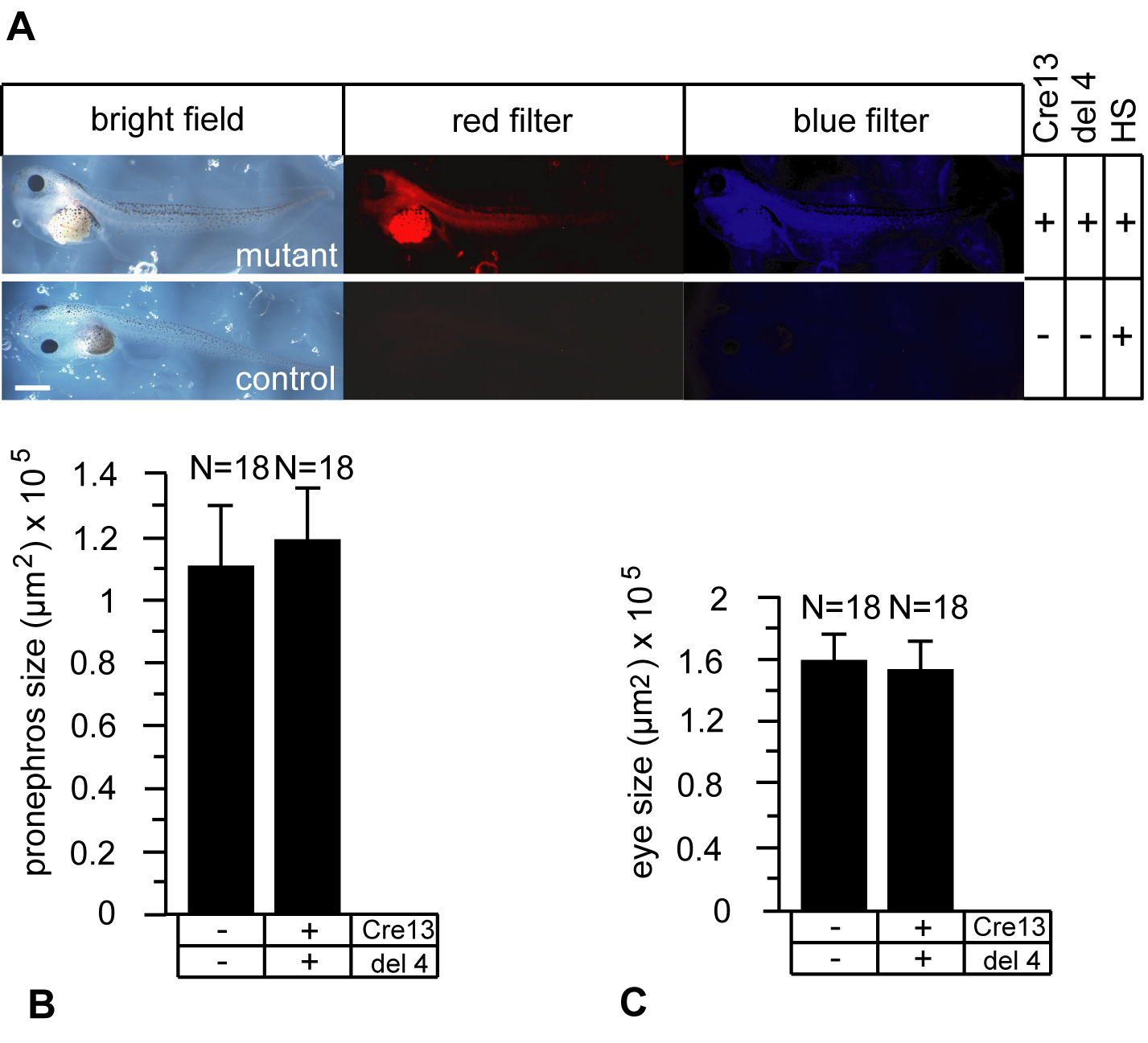

Supplement: Figure S3 — Effects of tailbud-stage overexpression of P328L329del HNF1β mutation on larval development. Representative larvae of crossings between heterozygous HSPCre13 males and heterozygous 328del4 females. The F1 offspring were heat-shocked for two hours at 34°C at tailbud stage (st. 25). A: Comparison of a larva with overexpressing the P328L329del HNF1β mutation with a non-transgenic control. Red fluorescence: HSPCre13 transgene. Blue fluorescence: 328del4 transgene. C and D: Quantitation of the average pronephric kidney (C) and eye sizes (D) in mutant and control animals. * = p<0.01 (Student's t-test). N refers to the number of single pronephri or eyes that were measured. Scale bar = 1 mm. (TIF) [file pone.0033522.s003.tif]
